# The Effect of GBV-C Infection on CD4 Count and Viral Loads in Patients Infected With HIV

**DOI:** 10.5812/kowsar.1735143x.4406

**Published:** 2012-01-20

**Authors:** Hossein Keyvani, Avid Mohammadi, Masoud Sabouri Ghannad, Mahboobeh Hajabdolbaghi

**Affiliations:** 1 School of Medicine, Tehran University of Medical Sciences, Tehran, IR Iran; 2 Keyvan Virology Laboratory, Tehran, IR Iran; 3 Department of Microbiology, Faculty of Medicine, Hamadan University of Medical Sciences, Hamadan, IR Iran; 4 AIDS Research Center, Tehran University of Medical Sciences, Tehran, IR Iran

**Keywords:** GB Virus C, HIV, Infection, CD4 Lymphocyte Count

## Abstract

**Background:**

The picture that has emerged from studies investigating HIV infected people with GBV-C viremia is that they have lower plasma HIV viral loads in comparison with HIV-positive people who did not have the GBV-C viremia.

**Objectives:**

Since GBV-C HIV coinfection has not been studied in Iran, we have designed a survey to study the outcomes of GBV-C infection on HIV infected individuals.

**Patients and Methods:**

We analyzed 78 serum samples from HIV-positive patients in Tehran. The HIV positive statue was confirmed by Western blot in our laboratory. Next we detected GBV-C RNA by RT nested-PCR and divided our patient into GBV-C positive and GBV-C negative groups. The final step was measuring the CD4 count and HIV viral load and comparing the means of the CD4 count and HIV viral load in HIV-infected individuals in the GBV-C positive and GBV-C negative groups.

**Results:**

We detected GBV-C RNA in 15 patients out of 78. The mean CD4 count was 607.13 compared to 415.87 in the GBV-C negative group and the difference was significant (P = 0.005). In contrast to the CD4 count there was no significant difference in HIV viral loads between HIV infected individuals in the GBV-C positive and GBV-C negative groups.

**Conclusion:**

Although there was no significant difference in the mean of the HIV viral load between the GBV-C positive and GBV-C negative groups, the significantly higher CD4 mean in the GBV-C positive group compared with the GBV-C negative group suggests a beneficial effect of this coinfection.

## 1. Background

GBV-C is a member of the Flaviviridae family and it is closely related to the hepatitis C virus (HCV) [1]. A GBV-C infection can persist for several years, but it usually causes no obvious clinical illness or death [2]. In healthy blood donors, GBV-C infection persistence occurs at rates of 1.8 % [3]. GBV-C infection clearance occurs in about 60% to 70% of immunocompetent GBV-C infected people with the appearance of anti-E2 viral glycoprotein antibodies [4][5]. Furthermore, GBV-C is a blood borne virus which can also be transmitted sexually, so a high prevalence of GBV-C infection is to be expected in human immunodeficiency virus (HIV) infected people (up to 35%) [3][6]. In 1998, Heringlake et al. described an association between GBV-C viremia and prolonged survival in HIV-GBV-C coinfected patients compared with HIV-infected subjects [7]. The results of some studies investigating the presence of GBV-C RNA in HIV-infected patients, showed that patients with GBV-C viremia had lower mortality rates, higher baseline CD4 T cell counts and also a slower rate of decline in the number of CD4 T cells [7][8][9][10][11][12]. In addition, the picture that has emerged from studies investigating HIV infected people with GBV-C viremia is that they have lower plasma HIV viral loads in comparison with HIV-positive people who did not have the GBV-C viremia [7][8][9][10][11][12]. However, some studies did not find these potentially positive effects [13][14][15]. To the best of our knowledge, in Iran the effects of HIV-GBV-C coinfection have not been widely evaluated and the prevalence of GBV-C in the HIV infected population has just been reported in our previous study [16]. The coinfection of HIV-GBV-C can be considered variously depending on the population under study, HIV and GBV-C genotypes might be different in various geographical regions of the world.

## 2. Objectives

The main aim of this study was to design a cross-sectional study to investigate HIV-GBV-C coinfection and its effects on HIV viral load and also CD4 T cell counts in coinfected patients in Tehran, the capital of Iran.

## 3. Patients and Methods

### 3.1. Study Population

The 78 patients involved in our cross-sectional study, were selected from the Imam Khomeini Hospital in Tehran, they were classified as HIV positive by an ELISA, and we confirmed their HIV infection using the Western blot method. Data such as sex, age, transmission routes and finally HCV or HBV coinfection were collected from the patients' files (Table 1). However, we were unable to follow up 7 patients out of the 78 for further information. The patients were between 5 and 54 years (mean 34.7 years) the group consisted of 87.5% males and 12.5% females. None of our patients were prescribed anti-HIV drugs.

**Table 1:** Patient Information

	**With GBV-C RNA**	**Without GBV-C RNA**	**P value**
Sex, No. (%)			
Male	13 (21)	49 (79)	0.67
Female	1 (11.1)	8 (88.9)	0.67
Age, y, No. (%)			
< 10	0 (0)	1 (100)	0.95
10 - 20	0 (0)	0 (0)	0.95
20 - 30	4 (26.7)	11 (73.3)	0.95
30 - 40	6 (18.2)	27 (81.8)	0.95
40 - 50	4 (22.2)	14 (77.8)	0.95
> 50	0 (0)	4 (100)	0.95
HIV transmission routes, %			
Intravenous drug use	83.3	35.3	NE a
Heterosexual contact	16.7	35.3	NE
Blood transmission	0	16.7	NE
Mother to infant	0	5.9	NE
Addict and Sexual	0	5.9	NE

^a^ Abbreviations: NE, not evaluated

### 3.2. Detection of GBV-C Viremia

Viral RNA was extracted from 100µl of serum or plasma using the acid guanidinium isothiocyanate-phenol chloroform method. For C-DNA synthesis, RT-PCR was performed at 42° C for 30 min. The C-DNA was stored at -20° C. Positive and negative controls were included in each run. Positive controls were provided from the Keyvan laboratory and their GBV-C genome was sequenced at the Seqlab, Germany.

### 3.3. Nested-PCR

Two sets of primers were used in nested-PCR.

External primers:

HG1: 5'GCCTATTGGTCAAGAGAGAC3'

HG2: 5'CACTATAGGTGGGTCTTAAG3'

Internal primers:

HG3: 5'GCGCACGGTCCACAGGTGTT3'

HG4: 5'GGGCGACGTGGACCGTACGT3'

The first PCR round was run for 30 cycles (94°C for 0.5 min, 55°C for 1.5 min and 72°C for 1.5 min). 10µl of product from the first PCR round was used as a template in the second PCR round. The second PCR round was for 35 cycles (94°C for 0.5 min, 55°C for 1.5 min and 72°C for 1.5 min). PCR products were separated by agarose gel electrophoresis and visualized by ethidium bromide staining.

### 3.4. HIV viral loads

Plasma HIV RNA load was quantified by a PCR (COBAS AMOLICOR HIV-1 Monitor kit, version 1.5; Roche Molecular Systems, Switzerland)

### 3.5. CD4 Cell count

CD4 cells count was performed in the Imam Khomeini Hospital at the same time as viral loads were analyzed in our laboratory.

### 3.6. Statistical analysis

Chi square and Man Whitney tests were used in comparing CD4 cells count and HIV viral loads between GBV-C positive and negative groups in HIV-infected persons. A P value < 0.05 was considered significant All statistical analysis was done by SPSS software version 15.

## 4. Results

Of the 78 patients investigated in our study the majority of patients were in the 30-40 years age group, and the major transmission route was through intravenous drug usage (Table 1). In 15 out of 78 patients, GBV-C RNA was detectable. Therefore, we divided our patients into two groups; GBV-C positive and GBV-C negative patients. Hepatitis C virus (HCV) and hepatitis B virus (HBV) coinfection were also considerations in these patients. Among the HIV-infected individuals, 12 patients were infected with HCV and one was also infected with HBV (Table 2). Among the HIV-HCV infected patients, 3 patients also had GBV-C viremia. GBV-C viremia was not detected in the only patient coinfected with HCV/HBV/HIV (Table 2). The mean value of HIV viral loads and CD4 cell counts were evaluated in GBV-C positive and GBV-C negative groups(Table 3). Comparing the HIV viral load means between the GBV-C positive and GBV-C negative groups in HIV positive patients showed no significant difference (P = 0.43). In contrast, the mean of the CD4 cell counts among GBV-C positive patients was significantly higher than in the GBV-C negative patients (P = 0.005).

**Table 2:** HCV/HBV Coinfection Statue in HIV-Infected Individuals

	**GBV-C Positive, No.**	**GBV-C Negative, No.**	**Total, No.**
HIV individuals with HCV coinfection	3	9	12
HIV individuals without HCV coinfection	12	53	65
HIV individuals with HCV/HBV coinfection	0	1	1

**Table 3:** CD4 Count and HIV Viral Load Means in GBV-C Positive and Negative Groups

	**GBV-C Positive**	**GBV-C Negative**	**P value**
CD4 count	607.13	415.87	0.005
HIV viral load	89480.73	92223.18	0.43

## 5. Discussion

The effect of a GBV-C coinfection on the disease progress in HIV-infected individuals is incompletely understood. Our patients were mainly between 30-40 years old and their predominant transmission route was intravenous drug usage, which is in agreement with previous reports investigating the prevalence of HIV in Iran [17][18]. In our cross-sectional study, the CD4 count was higher in the GBV-C positive group compared with the GBV-C negative group in the HIV-positive individuals, and a significant difference (P = 0.005) was observed. However, there was no significant difference in the HIV viral loads between the GBV-C positive and negative groups (P = 0.43) (table 3). Similar experiences have reported on the potential influence of GBV-C on CD4 counts [19][20][21] Taken together, Other researches demonstrate a significant association between the presence of GBV-C RNA in the serum of HIV-infected individuals and higher CD4 counts [19][20][21]. Hollingsworth et al. found levels of CD4 counts below 200 cell/μl in 25% of GBV-C positive patients compared with 75% in the GBV-C negative patients (P = 0.0382) [19]. In addition, the HIV-viral load tended to be lower, although it was not significant. Ibanez et al. and Bonacini et al. also reported a higher CD4 count in GBV-C positive patients, which was significant compared to CD4 counts in GBV-C negative patients. However, in the latter two studies, HIV viral loads were not considered. In contrast, Lau et al. Wooly et al. and Goubau et al. failed to show any association between the presence of GBV-C RNA and a higher CD4 count in GBV-C positive patients among HIV-infected individuals [22][23][24].

Part of our results obtained in this current research is consistent with the data already published. However, there are explanations for the different results obtained in our study compared to the data collected from other studies, which found positive effects or no significant effects for GBV-C HIV co-infection. The main noticeable explanation which needs to be considered is that, in most studies investigating GBV-C HIV coinfection, the investigators followed-up their patients for a period of time, in some cases over several years. Thus, they could measure CD4 counts and HIV viral loads at different periods through the years of follow-up. Also they could evaluate the progress of HIV-positive-individuals to AIDS, so they could compare the results they obtained during a period of time and also the mortality rate between GBV-C positive and GBV-C negative groups in HIV-positive individuals.

Our study was a preliminary survey to assess the GBV-C HIV coinfection statue in Iran. We did not have any information regarding GBV-C genotypes in Iran which might have a different effect on HIV in various other geographical regions. In addition follow-up was not possible because the patients were out of reach. This was a limitation that we faced during this stage of the study. As noted above, although the CD4 count mean was significantly higher in the GBV-C positive group, the HIV viral load mean did not show a significant difference between the GBV-C positive and negative groups. Other studies showed lower HIV viral loads in the plasma of HIV-positive people who had GBV-C viremia, so there is some discrepancy in the results from our research [7][8][9][10][11][12]. It is not unusual for different or even contradictory conclusions to be reported in the literature for similar subjects in the same field of research, as different results may be attributable to the experimental system used. In our study, although there was no significant difference between the mean of the HIV viral loads in the GBV-C positive and GBV-C negative groups, the mean of the CD4 count was significantly higher in the GBV-C positive patients in comparison with the GBV-C negative patients. Our study's results indicate a possible beneficial effect of a GBV-C coinfection with HIV.

Ongoing attempts will be undertaken to design more studies of patients infected with GBV-C and the effects of GBV-C persistent infections. As well as the genotyping of GBV-C, surveying the possible effects of other kinds of coinfections such as HCV and HBV on HIV-infected individuals and ultimately following-up the patients enrolling in such a project for a period of time, seem to be interesting ideas warranting further research in Iran.
